# Pathogen Transmission and Clinic Scheduling

**DOI:** 10.3201/eid1201.050349

**Published:** 2006-01

**Authors:** John R. Hotchkiss, David G. Strike, Philip S. Crooke

**Affiliations:** * University of Pittsburgh, Pittsburgh, Pennsylvania, USA;; † Regions Hospital, St. Paul, Minnesota, USA;; ‡ University of Minnesota, Minneapolis, Minnesota, USA;; § Vanderbilt University, Nashville, Tennessee, USA

**Keywords:** Outpatient clinic, mathematical model, infection control, computer simulation, dispatch

## Abstract

We developed a model of pathogen dissemination in the outpatient clinic that incorporates key kinetic aspects of the transmission process, as well as uncertainty regarding whether or not each incident patient is contagious. Assigning appointments late in the day to patients suspected of being infectious should decrease pathogen dissemination.

Pathogen dissemination within hospitals has been extensively analyzed (1). However, it has been the subject of far fewer investigations within the outpatient clinic. We developed a model of pathogen dissemination in the outpatient clinic and explored the anticipated effects of a system-based intervention (temporal segregation of suspected infectious and noninfectious patients) and an individual-based intervention (increased compliance with hand hygiene) on the risk that an uncontaminated patient will become contaminated during a clinic visit. An annotated copy of the model, as well as more detailed simulations and supporting material, may be obtained from the corresponding author.

## The Model

**Figure 1:**
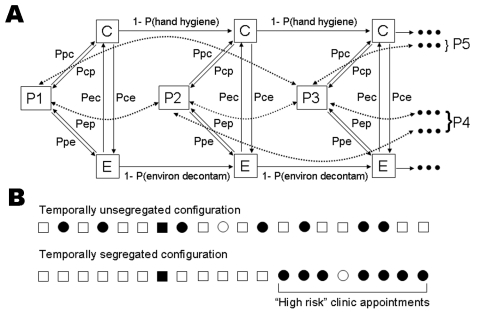
Schematic of model and segregation. A) Depiction of first 3 patient encounters. P1, P2, and P3, patients 1, 2, and 3; C, caregiver; E, environment. Arrows depict path (direction) of transmission if 1 participant in the interaction is infectious or contaminated. P(hand hygiene), probability that a contaminated caregiver will clear his or her contamination between patients; P(environ decontam), probability that a contaminated environment will be effectively decontaminated between patient visits. Direct patient-to-patient transmission is shown by dashed arrows. Because the model treats patient-to-patient transmission as a symmetrical process (the probability of transmission from patient to patient is identical regardless of which of the interacting patients is infectious), dashed arrows have 2 heads. B) Effects of different scheduling strategies. Solid circles, infectious high-risk patients; open circles, noninfectious high-risk patients; solid squares, infectious low-risk patients; open squares, noninfectious low-risk patients. Dots and arrows leading to P4 and P5 represent continuation of the chain of transmission. Ppc, probability of transmission from patient to caregiver; Pcp, probability of transmission from caregiver to patient; Pec, probability of transmission from environment to caregiver; Pce, probability of transmission from caregiver to environment.

We treat pathogen dissemination as a stochastic (chance-based) sequence of discrete encounters between incident patients (P) who are either infectious or noninfectious, a caregiver (C), and the environment (fomites, such as surfaces or waiting room magazines, [E]), each of which can be contaminated or uncontaminated. Four classes of encounter exist: 1) caregiver-patient, 2) caregiver-environment, 3) patient-environment, and 4) patient-patient. For each class of encounter, a user specifies a probability that a contaminated or infectious participant can transmit the pathogen to an uncontaminated participant (caregiver, environment [fomites], and surrounding patients). A contaminated caregiver can, in turn, contaminate subsequent patients (Figure 1). The contamination probabilities are not predicated on a specific mode of transmission but represent the gross probability that a contaminated or infectious participant will contaminate an uncontaminated participant during an encounter. With the exception of patient-to-patient transmission (see below) the contamination probabilities can be asymmetrical. Asymmetrical contamination probabilities allow consideration of droplet transmission; e.g., an infectious patient could cough on a caregiver, who then transfers the pathogen from his or her hands to the next patient.

An infectious patient can also contaminate the 4 patients surrounding him or her in the clinic queue (2 preceding and 2 following patients). The model does not incorporate patient-patient transmission by contaminated patients, given the relatively low frequency of intimate patient-to-patient physical contact in the clinic.

If contaminated, the caregiver may be decontaminated, e.g., by hand hygiene or pathogen attrition, with a specified probability after each patient visit. Similarly, a specified probability exists that the environment, if contaminated, will be decontaminated between visits. These 2 decontamination processes (for the caregiver and the environment) are independent. Both the caregiver and the environment can be recontaminated after decontamination. The contamination probabilities for transmission from a contaminated environment to a patient or the caregiver are fixed (i.e., not a function of the number of preceding infectious persons).

The model generates a random number for each class of encounter between potentially contaminated or infectious and uncontaminated participants during each clinic appointment slot. If this random number is less than the contamination probability specific to the class of interaction being considered, the uncontaminated member becomes contaminated during the encounter.

The user specifies the population prevalence of the pathogen. A screening instrument for classifying patients as high or low risk for being infectious is assumed to exist. We assume that the screening instrument can be applied before the patient's clinic visit (at the time of appointment scheduling). The screening instrument could comprise a symptom inventory, knowledge of recent travel, household exposure, membership in a known high-risk group, or a combination of these or other elements. Such screening instruments exist for methicillin-resistant Staphylococcus aureus, vancomycin-resistant enterococci, and severe acute respiratory syndrome (SARS) (2–5). The postulated instrument has a defined sensitivity and specificity; values that can be combined with the population prevalence of infectious patients to determine how many patients deemed at high risk or low risk are actually contagious.

Incident patients are assigned to appointment times following 1 of 2 protocols. In the baseline protocol, patients are randomly assigned appointments without regard to their risk status. In the segregated protocol, patients at high risk are assigned to appointments at the end of the clinic day (Figure 1). Clinics that segregate high-risk patients to later appointments might also adopt more stringent infection control strategies during the portion of the day populated by high-risk patients. Accordingly, the user can specify values for each contamination probability that differ between low-risk (early in the day) and high-risk (late in the day) clinic slots.

We modeled 1 day in a clinic in which 20 patients are seen by 1 caregiver. The model predicts the likelihood (risk) that a previously uncontaminated patient will become contaminated during his or her clinic visit. The model also predicts the risk that a patient who is classified as high-risk based on the screening instrument, but who is not infectious, will be contaminated in the segregated configuration.

**Figure 2:**
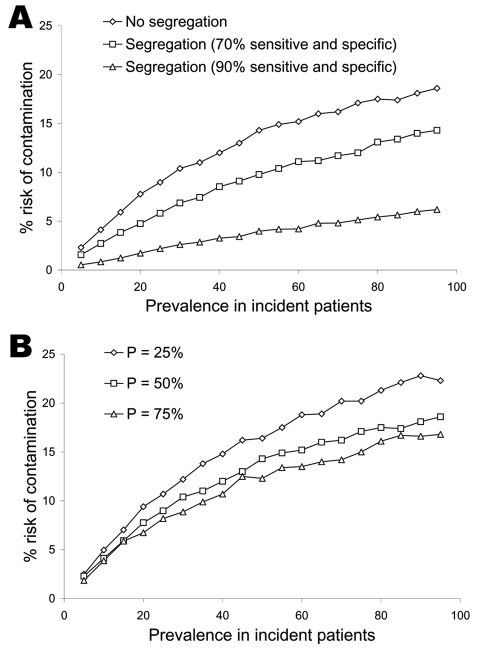
Risk that an uncontaminated patient will become contaminated during his or her clinic visit as a function of pathogen prevalence in incident patients and clinic infection-control practices. A) Predicted effects of temporally segregating patients at high risk of being infectious to appointments at the end of the clinic day, using a screening instrument that is either 70% sensitive and specific or 90% sensitive and specific. Transmission, hand hygiene, and environmental decontamination probabilities are as given in Table 1. B, Effects of varying levels of effective caregiver hand hygiene (25%, 50%, or 75%) on pathogen dissemination. All other inputs (probabilities of contamination) are identical to those in A. Each data point represents the mean of 2,000 simulations of a model day. An annotated copy of the model, as well as more detailed simulations and supporting material, may be obtained from the corresponding author.

Figure 2 illustrates the effect of temporally segregating patients deemed to be at high risk of being infectious to clinic appointments late in the clinic schedule (panel A) and the consequences of changes in caregiver hand-hygiene compliance (panel B). Relevant pathogen-contamination probabilities were arbitrarily fixed at 20%, a level that is reasonable, and possibly conservative, for a wide range of pathogens (Table 1) (6–14). Temporal segregation substantially decreases the risk for pathogen dissemination, an effect that is at least comparable in magnitude to that arising from changes in the likelihood of effective hand hygiene within the clinically relevant range (15).

**Table 1:** Interparticipant transmission and intraindividual transition probabilities used in simulations*

	Patient negative	Caregiver negative	Environment negative
Patient positive	0	P_PC_ = 0.2	P_PE_ = 0.2
Caregiver positive	P_CP_ = 0.2	P(hand hygiene) = 0.5	P_CE_ = 0
Environment positive	P_EP_ = 0.2	P_EC_ = 0.2	P(environ decont) = 0
Temporally adjacent patient positive	P_PP_ = 0	–	–

*P_PC_, probability of transmission from patient to caregiver; P_PE_, probability of transmission from patient to environment;  P_CP_, probability of transmission from caregiver to patient;  P(hand hygiene), probability that a contaminated caregiver will clear his or her contamination between patients;  P_CE_, probability of transmission from caregiver to environment;  P_EP_, probability of transmission from environment to patient;  P_EC_, probability of transmission from environment to caregiver;  P(environ decont), probability that contaminated environment will be decontaminated;  P_PP_, probability of transmission from patient to patient.

Table 2 addresses the ethical concern of the increase in contamination risk faced by noninfectious incident patients who are classified as high risk, using the same system inputs shown in Figure 2. The absolute risk for contamination faced by noninfectious but high-risk patients increases at all levels of prevalence and screening tool sensitivity and specificity; however, this increase does not exceed 6% (in the setting of a completely ineffective screening tool). More extensive analysis demonstrates that selectively deploying more aggressive infection-control practices to slots designated as high risk can lower the risk faced by noninfectious but nominally high-risk patients to below its value in the unsegregated configuration. These data are available from the corresponding author.

**Table 2:** Maximum absolute changes in contamination risk associated with temporal segregation

Prevalence (%)	Low-risk population		Noninfectious high-risk population	
	Maximum (%)	Minimum (%)	Maximum (%)	Minimum (%)
5	–2.1	–1	1	0.27
10	–4	–1.8	1.6	1.1
20	–6.8	–2.7	3.6	0
40	–10.7	–4.4	5.7	1

## Conclusions

The results do not address direct patient-to-patient pathogen transmission; such transmission does not change the qualitative predictions of the model. The model does not incorporate either the potential for the environmental pathogen load to increase over time or differences in host susceptibility. The potential for cross-transmission of additional pathogens that have a higher prevalence in the high-risk group is also not addressed. For example, a population of patients deemed at high risk of having influenza or SARS might also have a higher prevalence of other transmissible pathogens, such as Mycobacterium tuberculosis.

Improved hand hygiene, barrier precautions, patient use of hand sanitizers or facemasks, and avoidance of environmental fomites can each diminish dissemination risk. However, these measures depend on individual behavior, rendering them susceptible to implementation failure. Temporal segregation adds a system-based layer of protection to such behavior-based interventions, providing an additional barrier to pathogen dissemination that is not critically dependent on individual compliance. When additional data addressing pathogen transmission within the clinic become available (currently such data are sparse), mathematical models could help guide the allocation of resources required to support system-based infection-control measures. Moreover, numerical experimentation could help inform the design of infection-control strategies for pathogens that have transmission dynamics that are not yet well characterized.
